# CircRAPGEF5 Promotes the Proliferation and Metastasis of Lung Adenocarcinoma through the miR-1236-3p/ZEB1 Axis and Serves as a Potential Biomarker

**DOI:** 10.7150/ijbs.66770

**Published:** 2022-02-28

**Authors:** Huixin Zhou, Xiaolu Huang, Xiang Yang, Feng Jiang, Fanggui Shao, Wenjing Shi, Kate Huang, Jingjing Pan, Yan Zhang, Jie Chen, Yumin Wang

**Affiliations:** 1 Department of Clinical Laboratory, The First Affiliated Hospital of Wenzhou Medical University; Key Laboratory of Clinical Laboratory Diagnosis and Translational Research of Zhejiang Province, Wenzhou, 325000, China; 2 Department of Pathology, the First Affiliated Hospital of Wenzhou Medical University, Wenzhou, 325000, China; 3 Department of Intensive Care Unit, the First Affiliated Hospital of Wenzhou Medical University, Wenzhou, 325000, China

**Keywords:** lung adenocarcinoma, circRAPGEF5, exosomes, proliferation, metastasis

## Abstract

Lung adenocarcinoma (LAD) is a common malignancy; however, its underlying molecular mechanism is unclear. Circular RNAs (circRNAs) serve as significant cancer regulators. The overexpression of circRAPGEF5 in LAD tissues and cells indicated that it may be involved in promoting LAD progression. Analysis of 61 LAD tissues revealed that circRAPGEF5 was related to lymph node metastasis. Functionally, circRAPGEF5 promoted the proliferation, migration, invasion, and epithelial-mesenchymal transition of LAD cells *in vitro* and promoted LAD cells growth *in vivo*. Mechanistically, dual-luciferase reporter assays confirmed direct interaction of circRAPGEF5, miR-1236-3p, and ZEB1. miR-1236-3p was upregulated and ZEB1 expression reduced after circRAPGEF5 knockdown, and the proliferation, migration, and invasion of LAD cells was inhibited. circRAPGEF5 was significantly overexpressed in LAD cell exosomes, and co-culture experiments showed that exosomal circRAPGEF5 enhanced the metastatic ability of LAD cells. Further experiments found that serum exosomal circRAPGEF5 was overexpressed in LAD; moreover, the area under the receiver operator characteristic curve of exosomal circRAPGEF5 was superior to that of serum carcinoembryonic antigen (CEA). Jointly detected serum exosomal circRAPGEF5 and serum CEA had better diagnostic performance than when detected individually. Thus, exosomal circRAPGEF5 could promote the proliferation and metastasis of LAD via the miR-1236-3p/ZEB1 axis and serum exosomal circRAPGEF5 may serve as a promising biomarker for LAD.

## Introduction

Lung adenocarcinoma (LAD) is a common type of lung cancer with high heterogeneity, complex clinical responses, and poor prognosis 1. The primary factors responsible for the deterioration of LAD are a lack of preventive measures and early diagnosis, extremely variable tumor environment, multidrug resistance, and metastatic spread 2. Although LAD has been extensively researched, there remains insufficient development in the diagnostics and treatment of LAD. Invasion and metastasis are the main reasons for treatment failure of LAD. Epithelial-mesenchymal transition (EMT) occurs in the early stage of metastasis, in which epithelial cells acquire a mesenchymal phenotype 3, thereby increasing the capability of tumor metastasis.

Circular RNAs (circRNAs) have a closed-loop structure, which protects them from degradation by ribonuclease R (RNase R) 4 making them ideal to use as biomarkers 5. Growing evidence has shown that circRNAs are involved in tumor progression through various pathways, which when combined with the abovementioned advantages, are promising biomarkers and treatment targets 6. Studies have shown that circRNAs could function as sponges for miRNAs, thereby affecting the expression levels of target genes 7. For example, circTFRC, as the competing endogenous RNA (ceRNA) of miR-107, regulates TFRC mRNA expression and plays an important role in the pathogenesis and progression of bladder cancer 8. However, the role of circRNAs in the pathogenesis and progression of LAD remains unclear and needs to be further explored.

Exosomes, as a promising biomarker of circulatory diseases, have been gradually become known and proven to be a means of long-distance intercellular communication *in vivo* 9. When secreted into the recipient cell, exosomes can affect the function of receptor cells through proteins 10, mRNA/miRNA 11, 12 and DNA 13, 14. Exosomes carrying various functional molecules can cause deterioration of the tumor 15.

We found that hsa_circ_0001681 was overexpressed in tissues, cell lines, and exosomes of LAD using microarray and real-time quantitative PCR (RT-qPCR). Therefore, we speculated that hsa_circ_0001681 might play a significant role in LAD through exosomes. According to the circBase database, we found that hsa_circ_0001681 is derived from RAPGEF5 (chr7:22,330,793-22,357,656) and also named as circRAPGEF5. Importantly, circRAPGEF5 promotes papillary thyroid hyperplasia and metastasis 16. However, circRAPGEF5 is expressed at lower levels in renal cell carcinoma and inhibits tumor growth and metastasis 17. This is different from the results for thyroid cancer, which may be related to the tissue-specific expression of circRAPGEF5. As it is unclear whether circRAPGEF5 plays a regulatory role in LAD, we conducted this study to explore the biological function and molecular mechanism of circRAPGEF5 in LAD.

## Materials and methods

### Tissues and serum samples

Sixty-one samples of LAD and paired paracancerous tissues were collected from 29 male and 32 female patients at the First Affiliated Hospital of Wenzhou Medical University between August 2018 and August 2020. None of the had patients received chemotherapy or radiotherapy before sample collection. Serum specimens were obtained from 37 patients with LAD and 45 healthy participants. After the serum specimens were centrifuged at 2,000 × g for 30 min at 4 °C, the supernatant was removed and stored at -80 °C for later analysis. All samples were confirmed using histopathological examination, and informed consent was obtained from all the patients/participants. This study was approved by the Institutional Ethical Review Committee of the First Affiliated Hospital of Wenzhou Medical University.

### Cell culture

LAD cells (HCC827, A549, H1299) and normal human bronchial epithelial cells (BEAS-2B) were used in this study. A549, H1299 and HCC827 cells were maintained in RPMI-1640 medium (Gibco, MA, USA) containing 10% fetal bovine serum (FBS) (Gibco), BEAS-2B cells were kept in DMEM medium (Gibco) containing 10% FBS, and incubated at 37 °C with 5% CO_2_. All cultures were routinely tested, and no mycoplasma or fungal contamination was detected.

### Transfection

The lentiviral vector containing the OE-circRAPGEF5 (circRAPGEFF5 overexpression) sequence and OE-NC (control of the OE group) were purchased from Genechem (Shanghai, China). A549 and H1299 cells were infected with the lentiviral vectors in an infection enhancement solution and replaced with RPMI-1640 complete medium after 12 h. Fluorescence expression was observed 3 d later. Stable cell lines were screened with puromycin (Solarbio, Beijing, China) at a final concentration of 2 μg/mL. KD-circRAPGEFF5 (circRAPGEF5 knockdown) was performed using antisense oligonucleotides, miR-1236-3p mimic, miR-1236-3p inhibitor, shZEB1 (ZEB1 knockdown), and matched KD-NC, mimic NC, inhibitor NC, and shNC were provided by RiboBio (Guangzhou, China) and transfected at a final concentration of 50 nM. Plasmid pcDNA-ZEB1 and control vector pcDNA-3.1 were purchased from Suzhou Emax Biotechnology Company (Suzhou, China), and transfected with 500 ng of the plasmid in 12-well plates. Lipofectamine 3000 (Invitrogen, Shanghai, China) was used for transfection.

### Extraction and identification of exosomes

Extraction of cell exosomes: When the cells grew to 70‒80% confluence, the original culture medium was replaced with exosomal-free serum medium, and the cell supernatant was collected after culturing for 48 h. The collected cell supernatant was centrifuged at 500 × g, 4 °C, 10 min; 2,000 × g, 4 °C, 20 min; 10,000 × g, 4 °C, 30 min and filtered using a 0.22 μm filter membrane (Millipore, MA, USA); the filtrate was centrifuged at 120,000 × g, 4 °C, 2 h for the separation of exosomes, and then washed with PBS. Extracted exosomes could be used directly or stored in a refrigerator at -80 °C.

Extraction of serum exosomes: The serum was diluted with the same amount of PBS, centrifuged at 2,000 × g, 4 °C, 30 min; 12,000 × g, 4 °C, 45 min, and then the supernatant was collected and filtered using a 0.22 μm filter membrane; the filtrate was centrifuged at 120,000 × g, 4 °C, 2 h for the separation of exosomes. After centrifugation, exosomes were washed with PBS and purified repeatedly through ultracentrifugation. Extracted serum exosomes could be used directly or stored in a refrigerator at -80 °C.

Transmission electron microscope (TEM) observation: PBS solution containing exosomes was absorbed, dropped on the clean surface of the parafilm, and the carbon film copper mesh was carefully suspended on the exosome droplets for 10 min, dried, and then 50 μl 3% phosphotungstic acid solution was added to the exosomes. The exosomes were dyed with 3% phosphotungstic acid dye solution for 5 min and then observed using a TEM and photographed (Hitachi, Tokyo, Japan).

Nanoparticle tracking analysis (NTA): PBS solution containing exosomes was absorbed, diluted with PBS if necessary, and exosome samples were drawn into the sample pool and tested on the machine according to the manufacturer's instructions. All the samples had the same detection threshold.

Western blot: Detection of exosomal marker proteins such as CD9, CD63, and TSG101 and the negative marker protein calnexin (all 1:1000, Protiontech, IL, USA).

### Total RNA extraction and RT-qPCR

An RNA extraction kit (Invitrogen, CA, USA) was used to extract total RNA from tissues, exosomes, and cell lines. The quality and concentration of RNA were tested using NanoDrop 2000 (Thermo, Shanghai, China). Before reverse transcription of circRAPGEFF5, RNA samples were divided into two uniform parts: one part was treated with RNase R (Geneseed Biotech, Guangzhou, China) at 37 °C for 10 min, and the other part was treated with an equal amount of RNase-free ddH_2_O without RNase R. miDETECT A Track^TM^ miRNA qPCR Kit (RiboBio) was used to reverse transcribe the miRNAs. Finally, RT-qPCR was performed using an ABI Q5 PCR instrument (Applied Biosystems, CA, USA). β-actin and U6 were used as internal references. Serum exosomal circRAPGEF5 expression analysis by the 2^-ΔCt^ method, the -ΔCt method was used for tissue samples, and other results were analyzed by the 2^-ΔΔCt^ method. The primer sequences used are listed in Supplementary Table S1.

### Cell Counting Kit-8 assay

A total of 2000 cells/well were inoculated into a 96-well plate. After culturing for 24 h, 10 μl Cell Counting Kit - 8 (CCK-8) was added (Dojindo, Japan). After incubation for 1 h, the absorbance was measured at 450 nm using a microplate reader (SpectreMax, MD, USA). This step was performed once a day after 24 h of plate laying, repeated for 4 d, accurately recorded the measured absorbance value, and drew the cell growth curve.

### Transwell migration and invasion assays

Transwell pore polycarbonate membrane inserts (Corning, pore size 8 μm, MD, USA) were used, and a layer of Matrigel (Corning) was coated on the insert during the invasion experiment. Cells were inoculated inside the chamber with or without exosome treatment. The concentration of exosomes was 50 μg/mL 18, 19. The upper chamber used RPMI-1640 medium, and the lower chamber used RPMI-1640 medium containing 20% FBS medium with or without exosomes. After 48 h of culturing, the cells were fixed and stained. Finally, the residual crystal violet was washed away with water. Cells that passed through the chamber were counted.

### Clone formation assay

Six hundred cells/well were inoculated into a six-well plate, cultured for 14 d, and then fixed and stained to observe the formation of cell colonies. The standard used more than 50 cell clusters as a cell clone. The number of cell clones was compared to determine the cell clone formation ability.

### Flow cytometry to detect cell apoptosis

An Apoptosis Detection Kit (KeyGEN, Nanjing, China) was used to detect cell apoptosis. After washing the cells twice with PBS, the cell pellet was resuspended in 500 μl Binding buffer, and 5 μl Annexin V-APC and 5 μl 7-AAD dye solution, the reaction proceeded for 5-15 min in the dark, and tested on a flow cytometer (Beckman, CA, USA).

### Western blotting

Cells were inoculated into six-well plates for protein sample extraction, and the exosomes extracted by ultracentrifugation could be used directly for protein extraction. A protein quantification kit (Beyotime, Shanghai, China) was used to adjust the protein sample load to 30 μg. After electrophoresis, transfer membrane, and blocking with 5% skim milk, protein bands were incubated with primary antibodies against ZEB1 (1:800, Protiontech), E-cadherin (1:5000, Protiontech), N-cadherin (1:2000, Protiontech), vimentin (1:2000, Protiontech), snail (1:800, Protiontech), slug (1:800, Protiontech), and β-actin (1:1000, Protiontech), and incubated with Horseradish peroxidase (HRP)-conjugated secondary goat anti-rabbit antibodies (1:2000, Beyotime). The enhanced chemiluminescence (ECL) reagent (Thermo) was used to observe protein expression using a gel imaging analysis system (Bio-Rad, CA, USA). β-actin was used as an internal reference.

### Separation of nucleus and cytoplasm

A Nuclear/Cytosol Fractionation Kit (BioVision, CA, USA) was used to localize circRAPGEF5, U6 for nuclear part self-inspection, and GAPDH for cytoplasmic part self-inspection. The primer sequences used are listed in Supplementary Table S1.

### Xenografts in mice

All animal procedures were approved by the Laboratory Animal Ethics Committee of the First Affiliated Hospital of Wenzhou Medical University (WYYY-AEC-2021-310). Twelve BALB/c nude mice(Zhejiang Weitong Lihua Laboratory Animal Technology Co., Ltd. Hangzhou, China) were randomly divided into two groups (six in each group), namely A549 OE-NC and A549 OE-circRAPGEF5 groups. The cell density was adjusted to 2.5 × 10^7^ cells/mL, and then the nude mice were injected with cell suspension subcutaneously into their right axilla to establish subcutaneous tumor models. Each nude mouse was injected with 200 μl ( 5 × 10^6^ cells). After the tumor was formed, the tumors were observed every 2 d, and the weight of the mice and tumor volume were measured. The volume calculation formula was (length × width^2^)/2. Twenty-four days later, the nude mice were sacrificed via excessive anesthesia, and the tumors were excised, photographed, and weighed.

### Dual-luciferase reporter assay

We constructed wild-type (WT) and mutated (Mut) sequences containing miR-1236-3p binding sites and cloned them into a dual-luciferase reporter plasmid containing the pmiRGLO promoter to obtain pmiRGLO-circRAPGEF5-WT/Mut and pmiRGLO-ZEB1-3' UTR-WT/Mut dual luciferase reporter vectors. A549 cells were inoculated into 24-well plates and cultured for 24 h. miR-1236-3p mimic or mimic NC was co-transfected with the constructed reporter vector pmiRGLO-circRAPGEF5-WT/Mut or pmiRGLO-ZEB1-3UTR-WT/Mut for 48 h. Finally, the dual-luciferase reporter analysis system kit (Promega, WI, USA) was used to evaluate the relative luciferase activity.

### Statistical analysis

GraphPad Prism 8 and SPSS 22.0 were used to analyze results. Unpaired *t* tests were utilized to determine the difference between the two groups, the paired *t* tests analyzed the difference between LAD tissues and paired paracancerous tissues, and the correlation between circRAPGEF5 expression and clinical features of LAD was analyzed by x^2^ tests. Pearson correlation analysis was used to analyze the correlation. Analyze the area under the receiver operator characteristic (ROC) curve (AUC) to determine the sensitivity and specificity of the test. A value of *P* < 0.05 was considered statistically significant. Data were expressed as the mean ± standard deviation of three independent tests.

## Results

### High expression and characteristics of circRAPGEF5 in LAD

**Figure 1:**
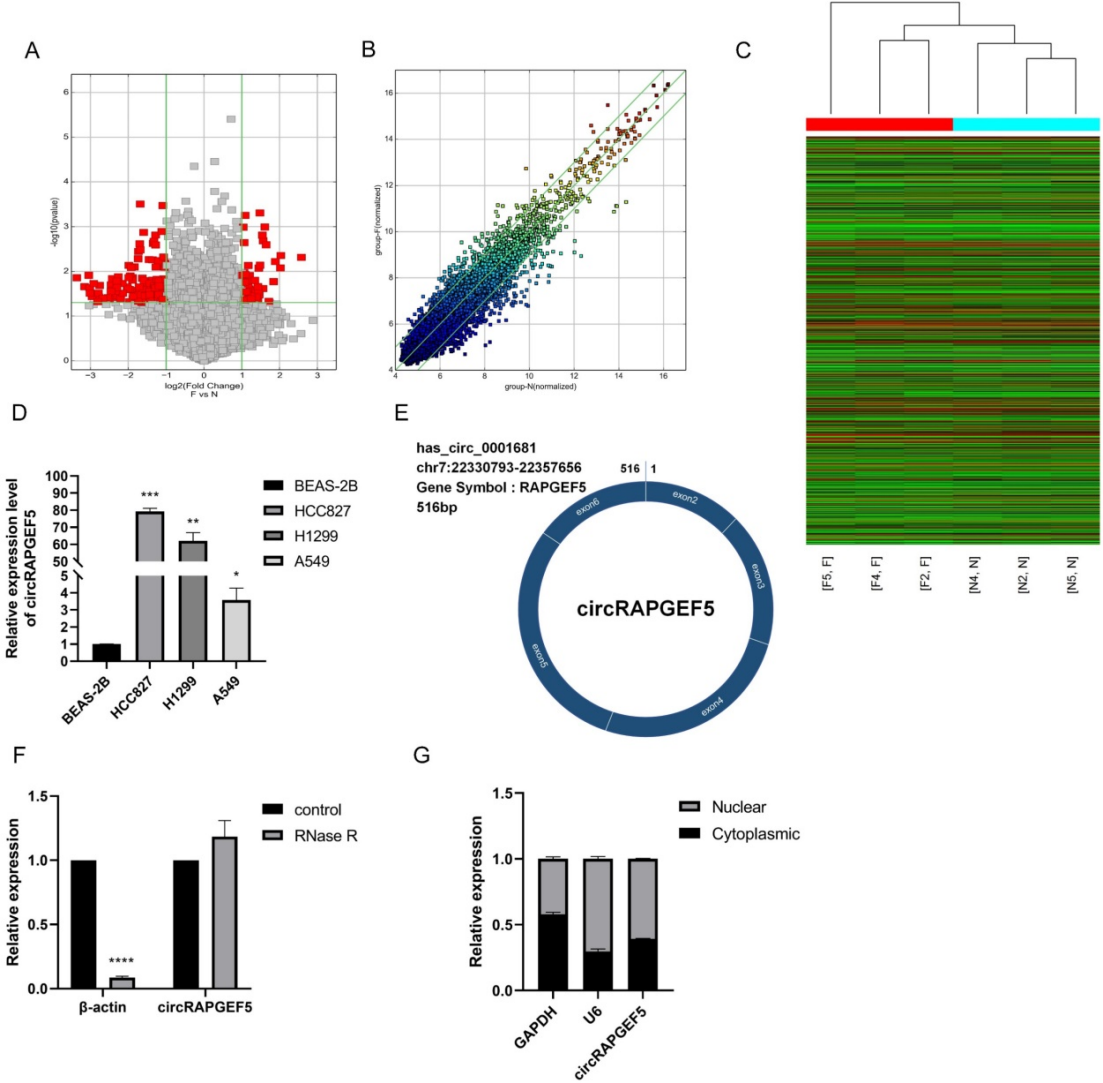
High expression and characteristics of circRAPGEF5 in LAD. The volcano map (A), scatter plot (B) and heat map (C) showed the circRNAs differential expression in lung adenocarcinoma (LAD) tissues and paired paracancerous tissues. (D) circRAPGEF5 expression in normal human bronchial epithelial cells (BEAS-2B) and LAD cells (A549, H1299 and HCC827) was detected by RT-qPCR. (E) The figure showed the ring structure of circRAPGEF5. (F) The expression of circRAPGEF5 after Ribonuclease R (RNase R) treatment (2 U/μg RNA, 10 min) was tested by RT-qPCR. (G) The localization of circRAPGEF5 was analyzed by RT-qPCR. *P < 0.05, **P < 0.01, ***P < 0.001, ****P < 0.0001.

We compared circRNA expression differences between LAD and paired paracancerous tissues using high-throughput microarrays. After identification, there were 57 upregulated and 149 downregulated circRNAs in the LAD tissues. Among them, hsa_circ_0001681 (named circRAPGEF5) had the highest expression (*P* < 0.01, Fig. 1A‒C; Supplementary Table S2). We found that circRAPGEF5 was overexpressed in LAD cells (Fig. 1D). CircBase database (http://www.circbase.org/) shows that circRAPGEF5 is a cyclization of five exons, with a length of 516 bp, located on chromosome 7 (Fig. 1E).

After RNase R treatment, the β-actin mRNA expression level decreased significantly, while the expression of circRAPGEF5 was not affected by RNase R treatment, indicating that circRAPGEF5 was highly stable (Fig. 1F) and confirming the circular structure of circRAPGEF5. Finally, we found that circRAPGEF5 was distributed in both the nucleus and cytoplasm, 60.75% and 39.25%, respectively, with nuclear and cytoplasmic separation technology (Fig. 1G).

### circRAPGEF5 promotes proliferation of LAD cells *in vitro* and *in vivo*

**Figure 2:**
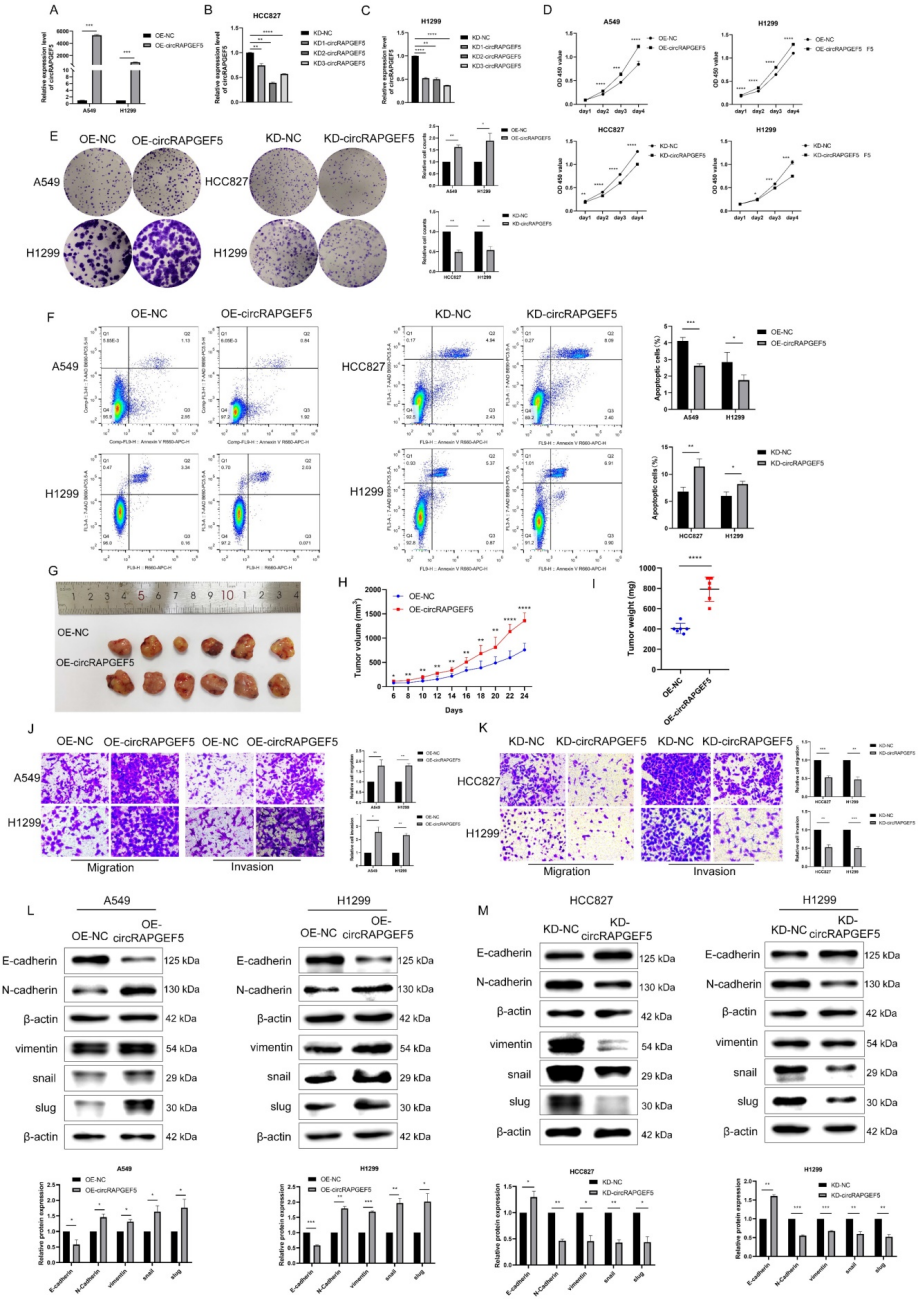
circRAPGEF5 promotes proliferation, migration, invasion and EMT process of LAD cell lines. (A) circRAPGEF5 expression in A549 and H1299 cells transfected with OE-circRAPGEF5 vector was detected by RT-qPCR. B, C, The transfection efficiency of KD-circRAPGEF5 (KD-circRAPGEF5: circRAPGEF5 knockdown; KD-NC: negative control of KD group; 50 nM) was detected by RT-qPCR in HCC827 (B) and H1299 (C) cells. (D) Cell Counting Kit-8 (CCK-8) assays detected proliferation of circRAPGEF5 overexpression and knockdown in LAD cells. (E) The colony formation assays detected clone formation ability of circRAPGEF5 overexpression and knockdown in LAD cells. (F) Flow cytometry analyzed apoptosis rates of circRAPGEF5 overexpression and knockdown in LAD cells. (G) Representative images of the xenograft after 24 d inoculation. H, I, Growth curve (H) and weight (I) of xenograft in nude mice. J, K, Transwell analysis detected migration and invasion of circRAPGEF5 overexpression (J) and knockdown (K) in LAD cells. L, M, Epithelial-mesenchymal transition (EMT) marker proteins expression in circRAPGEF5 overexpression (L) and knockdown (M) cells was tested. *P < 0.05, **P < 0.01, ***P < 0.001, ****P < 0.0001.

To investigate the roles of circRAPGEF5 in LAD cell lines, we first successfully constructed OE-circRAPGEF5 and KD-circRAPGEF5 cell lines (Supplementary Fig. S1; Fig. 2A‒C). KD2-circRAPGEF5 was significantly downregulated in HCC827 and H1299 cells; therefore, we used KD2-circRAPGEF5 to knock down circRAPGEF5 expression.

circRAPGEF5 overexpression promoted the proliferation and colony formation of A549 and H1299 cells, while circRAPGEF5 knockdown inhibited these effects in HCC827 and H1299 cells (Fig. 2D, E). Apoptosis assays showed that OE-circRAPGEF5 cells decreased the apoptotic ability, but KD-circRAPGEF5 cells increased the apoptotic ability (Fig. 2F). To study the effect of circRAPGEF5 on LAD tumor growth *in vivo*, we injected stably overexpressing circRAPGEF5 A549 cells into the subcutaneous axilla of nude mice and found that the volume of tumors in the circRAPGEF5 overexpression group was significantly larger than that in the control group (Fig. 2G). In addition, overexpression of circRAPGEF5 promoted tumor growth (Fig. 2H), and the tumor weight in the circRAPGEF5 overexpression group was significantly higher than that in the control group (Fig. 2I). In summary, the above results demonstrated that circRAPGEF5 promoted LAD cell proliferation *in vitro* and *in vivo*.

### circRAPGEF5 promotes migration, invasion, and EMT of LAD cell lines

The role of circRAPGEF5 in the metastasis of LAD cells was evaluated. We found that OE-circRAPGEF5 cells increased migration and invasion (Fig. 2J), while KD-circRAPGEF5 cells inhibited these effects (Fig. 2K). Moreover, EMT was assessed using western blotting in LAD cells. Epithelial cell marker E-cadherin expression was decreased, and mesenchymal cell markers (N-cadherin, vimentin, snail, and slug) were increased after circRAPGEF5 overexpression (Fig. 2L). In contrast, circRAPGEF5 knockdown cells showed the opposite results (Fig. 2M). The above results confirmed that circRAPGEF5 induced mesenchymal transformation, thereby promoting the migration, invasion, and EMT of LAD cells.

### circRAPGEF5 functions as a sponge of miR-1236-3p to promote LAD development

**Figure 3:**
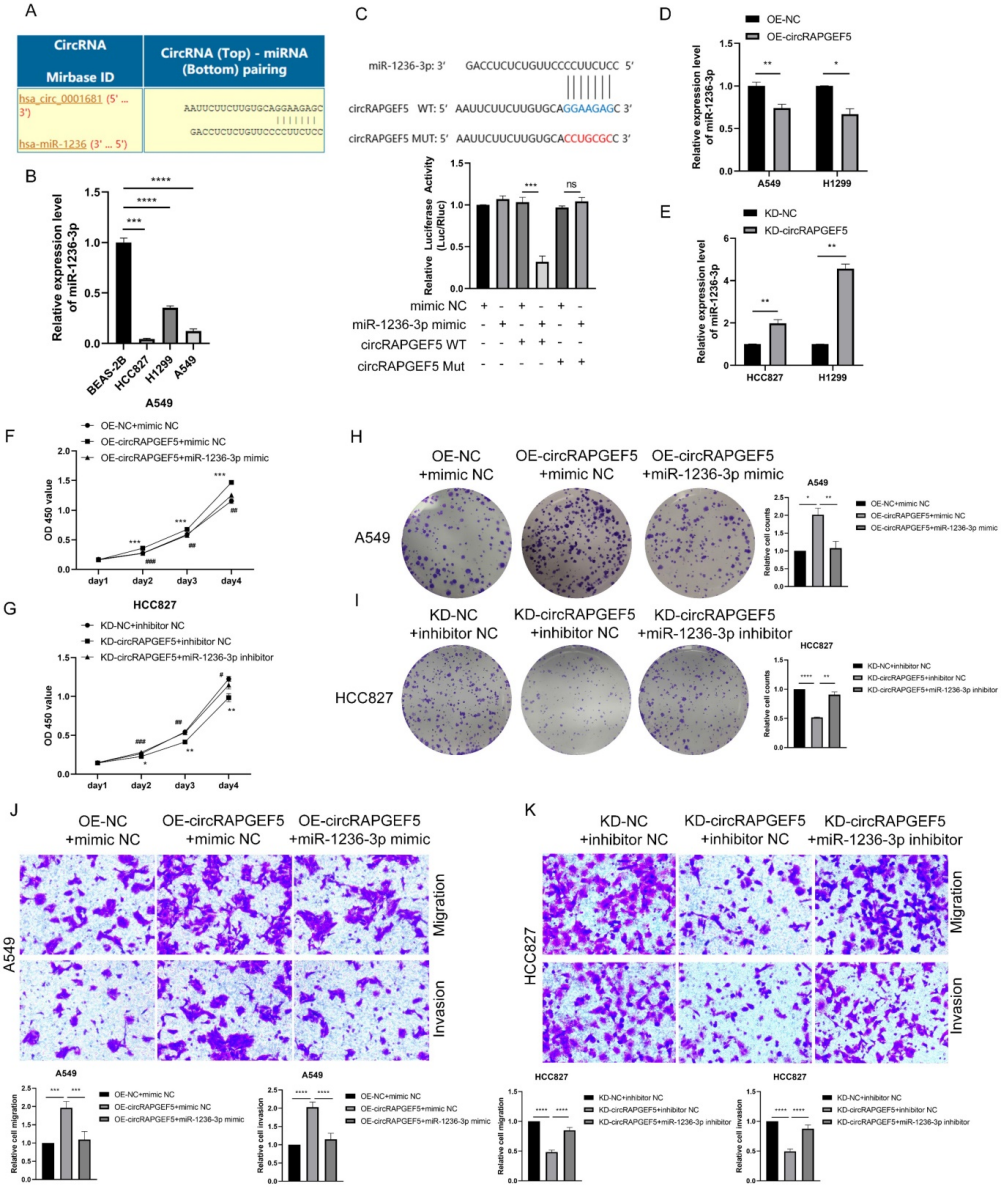
circRAPGEF5 functions as a sponge of miR-1236-3p to promote LAD development. (A) Network prediction diagram of circRAPGEF5 and miR-1236-3p. (B) miR-1236-3p expression in BEAS-2B cells and LAD cells was detected by RT-qPCR. (C) The luciferase activity was analyzed after co-transfection of miR-1236-3p mimic or mimic NC with the constructed reporter vector pmiRGLO-circRAPGEF5-WT/Mut. D, E, miR-1236-3p expression after overexpression (D) or knockdown (E) of circRAPGEF5 in LAD cells was analyzed by RT-qPCR. F, G, CCK-8 assays evaluated proliferation of transfected A549 (F) and HCC827 (G) cells. H, I, The colony formation assays evaluated clone formation ability of transfected A549 (H) and HCC827 (I) cells. J, K, Transwell analysis detected migration and invasion of transfected A549 (J) and HCC827 (K) cells. ns: no significance, *P < 0.05, **P < 0.01, ***P < 0.001, ****P < 0.0001 versus OE-NC+mimic NC or KD-NC+inhibitor NC, ^#^*P* < 0.05, ^##^*P* < 0.01, ^###^*P* <0.001, ^####^*P* < 0.0001 versus OE-circRAPGEF5+mimic NC or KD-circRAPGEF5+inhibitor NC.

The Circular RNA Interactome website (https://circinteractome.nia.nih.gov) was used to predicted potential miRNAs of circRAPGEF5, and it was found that miR-1236-3p may interact with circRAPGEF5 (Fig. 3A). In addition, miR-1236-3p inhibits the migration and invasion of LAD 20. We found that miR-1236-3p expression was significantly lower in LAD cells than that in BEAS-2B cells (Fig. 3B). Subsequently, the dual-luciferase reporter assay showed that the relative luciferase activity of circRAPGEF5 WT co-transfected with miR-1236-3p mimic was significantly lower than that of the other groups (Fig. 3C). In addition, we found that in the circRAPGEF5 overexpression group, miR-1236-3p was significantly downregulated (Fig. 3D), while miR-1236-3p was significantly upregulated in the circRAPGEF5 knockdown group (Fig. 3E). The above results indicated that circRAPGEF5 could directly bind to miR-1236-3p.

We further explored the interaction between circRAPGEF5 and miR-1236-3p, and found that co-transfection of miR-1236-3p mimic dramatically decreased the proliferation, migration, and invasion induced by circRAPGEF5 in A549 cells (Fig. 3F, H, J). In contrast, inhibition of miR-1236-3p could rescue the inhibition of proliferation, migration, and invasion by downregulating circRAPGEF5 in HCC827 cells (Fig. 3G, I, K). In summary, these results further confirmed the interaction between miR-1236-3p and circRAPGEF5 and that the tumor promoting effect of circRAPGEF5 could be reversed by miR-1236-3p.

### ZEB1 is a major target of circRAPGEF5 to promote LAD development

**Figure 4:**
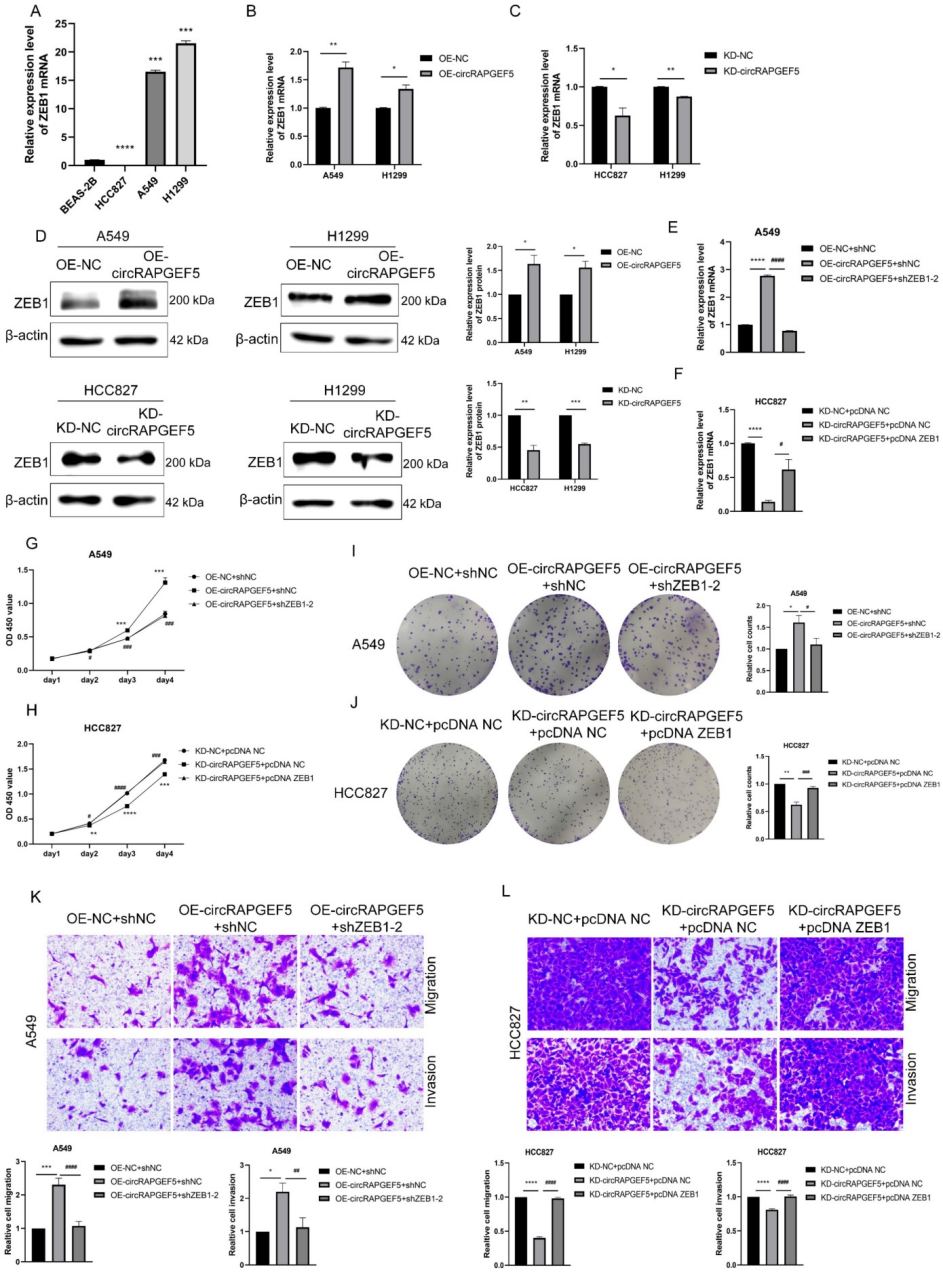
ZEB1 is a major target of circRAPGEF5 to promote LAD development. (A) ZEB1 expression in BEAS-2B cells and LAD cells was analyzed. B, C, ZEB1 expression after overexpression (B) or knockdown (C) of circRAPGEF5 in LAD cells was analyzed by RT-qPCR. (D) ZEB1 protein expression after overexpression or knockdown of circRAPGEF5 was tested. E, F, After OE-circRAPGEF5 were co-transfected with shZEB1-2 (ZEB1 knockdown, 50 nM) (E) and KD-circRAPGEF5 were co-transfected with pcDNA ZEB1 (ZEB1 overexpression, 500 ng/12-well plates) (F), the expression of ZEB1 was tested by RT-qPCR. G, H, CCK-8 assays evaluated proliferation of transfected A549 (G) and HCC827 (H) cells. I, J, The colony formation assays evaluated clone formation ability of transfected A549 (I) and HCC827 (J) cells. K, L, Transwell analysis detected migration and invasion ability of transfected A549 (K) and HCC827 (L) cells. *P < 0.05, **P < 0.01, ***P < 0.001, ****P < 0.0001 versus OE-NC+shNC or KD-NC+pcDNA NC, ^#^*P* < 0.05, ^##^*P* < 0.01, ^###^*P* < 0.001, ^####^*P* < 0.0001 versus OE-circRAPGEF5+shNC or KD-circRAPGEF5+pcDNA NC.

We confirmed that circRAPGEF5 is involved in promoting the EMT process. ZEB1, as a pivotal transcription factor for EMT 21, might be a potential target of circRAPGEF5. Compared with BEAS-2B cells, ZEB1 was upregulated in A549 and H1299 cells and downregulated in HCC827 cells (Fig. 4A). Subsequently, ZEB1 expression was found to be upregulated or downregulated in circRAPGEF5 overexpression or knockdown cells using RT-qPCR (Fig. 4B, C) and western blotting (Fig. 4D).

Furthermore, co-transfection with shZEB1 dramatically decreased the circRAPGEF5-upregulated ZEB1 expression in A549 cells (Fig. 4E), as well as proliferation, migration, and invasion capabilities (Fig. 4G, I, K). However, overexpression of ZEB1 rescued the KD-circRAPGEF5-inhibited ZEB1 expression in HCC827 cells (Fig. 4F), and the proliferation, migration, and invasion abilities could be reversed by ectopic overexpression of ZEB1 (Fig. 4H, J, L). In short, the above results indicated that ZEB1 was a major target for circRAPGEF5 to play tumor-promoting roles in LAD.

### ZEB1 promotes proliferation, migration, and invasion of LAD cell lines

To verify the role of ZEB1 in LAD cell lines, we transfected ZEB1 overexpression (pcDNA ZEB1) and knockdown (shZEB1) cells in A549 and H1299 cells, and their negative controls (pcDNA NC and shNC). Compared with their control cells, ZEB1 expression had significant differences between ZEB1 overexpression and knockdown (Supplementary Fig. S2A, B). In the ZEB1 knockdown group, shZEB1-2 knocked down ZEB1 expression in both A549 and H1299 cells, therefore, shZEB1-2 was used for ZEB1 knockdown in the next experiment.

ZEB1 overexpression promoted proliferation and colony formation and inhibited apoptosis, while ZEB1 knockdown produced the opposite results (Supplementary Fig. S2C‒E). High expression of ZEB1 promoted migration and invasion, while ZEB1 knockdown inhibited these effects (Supplementary Fig. S2F, G). These results demonstrated that ZEB1 promoted the proliferation, migration, and invasion of LAD cell lines.

### circRAPGEF5 promotes LAD proliferation and metastasis through the miR-1236-3p/ZEB1 axis

**Figure 5:**
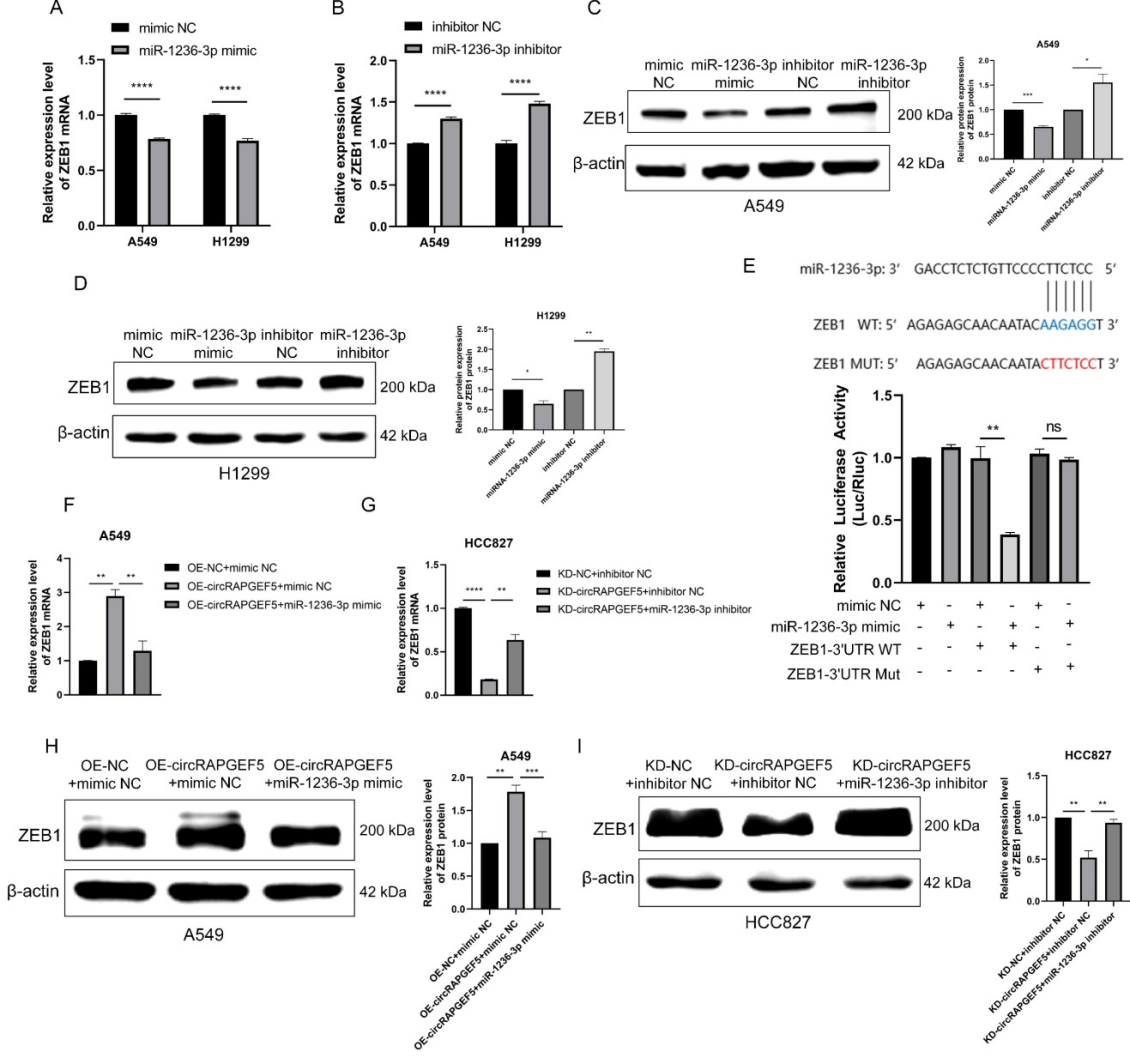
circRAPGEF5 promots LAD proliferation and metastasis through the miR-1236-3p/ZEB1 axis. A-D, ZEB1 expression after overexpression or inhibition of miR-1236-3p in LAD cells was analyzed by RT-qPCR (A, B) and western blotting (C, D). (E) The luciferase activity was analyzed after co-transfection of miR-1236-3p mimic or mimic NC with the constructed reporter vector pmiRGLO-ZEB1-3′UTR-WT/ Mut. F, H, ZEB1 expression after co-transfection of OE-circRAPGEF5 with miR-1236-3p mimic in A549 cells by RT-qPCR (F) and western blotting (H). G, I, ZEB1 expression after co-transfection of KD-circRAPGEF5 with miR-1236-3p inhibitor in HCC827 cells by RT-qPCR(G) and western blotting (I). ns: no significance, *P < 0.05, **P < 0.01, ***P < 0.001, ****P < 0.0001.

Studies have reported that miR-1236-3p regulates ZEB1 expression in the LAD 20. Therefore, we speculated that circRAPGEF5 may exert its function through the miR-1236-3p/ZEB1 axis. We found that ZEB1 expression was downregulated or upregulated in miR-1236-3p mimic or inhibitor cells using RT-qPCR (Fig. 5A, B) and western blotting (Fig. 5C, D). Subsequently, the dual-luciferase reporter assay showed that the relative luciferase activity of ZEB1 WT with miR-1236-3p mimic group was significantly lower than that of the other groups (Fig. 5E), confirming the interaction between miR-1236-3p and ZEB1.

In addition, we found that co-transfection of miR-1236-3p mimic dramatically decreased circRAPGEF5-upregulated ZEB1 expression in A549 cells (Fig. 5F, H). In contrast, inhibition of miR-1236-3p rescued the KD-circRAPGEF5-inhibited ZEB1 expression in HCC827 cells (Fig. 5G, I). In summary, the above results demonstrated the dominant role of the miR-1236-3p/ZEB1 axis in the proliferation and metastasis of LAD.

### circRAPGEF5, miR-1236-3p and ZEB1 expression in LAD tissues

**Figure 6:**
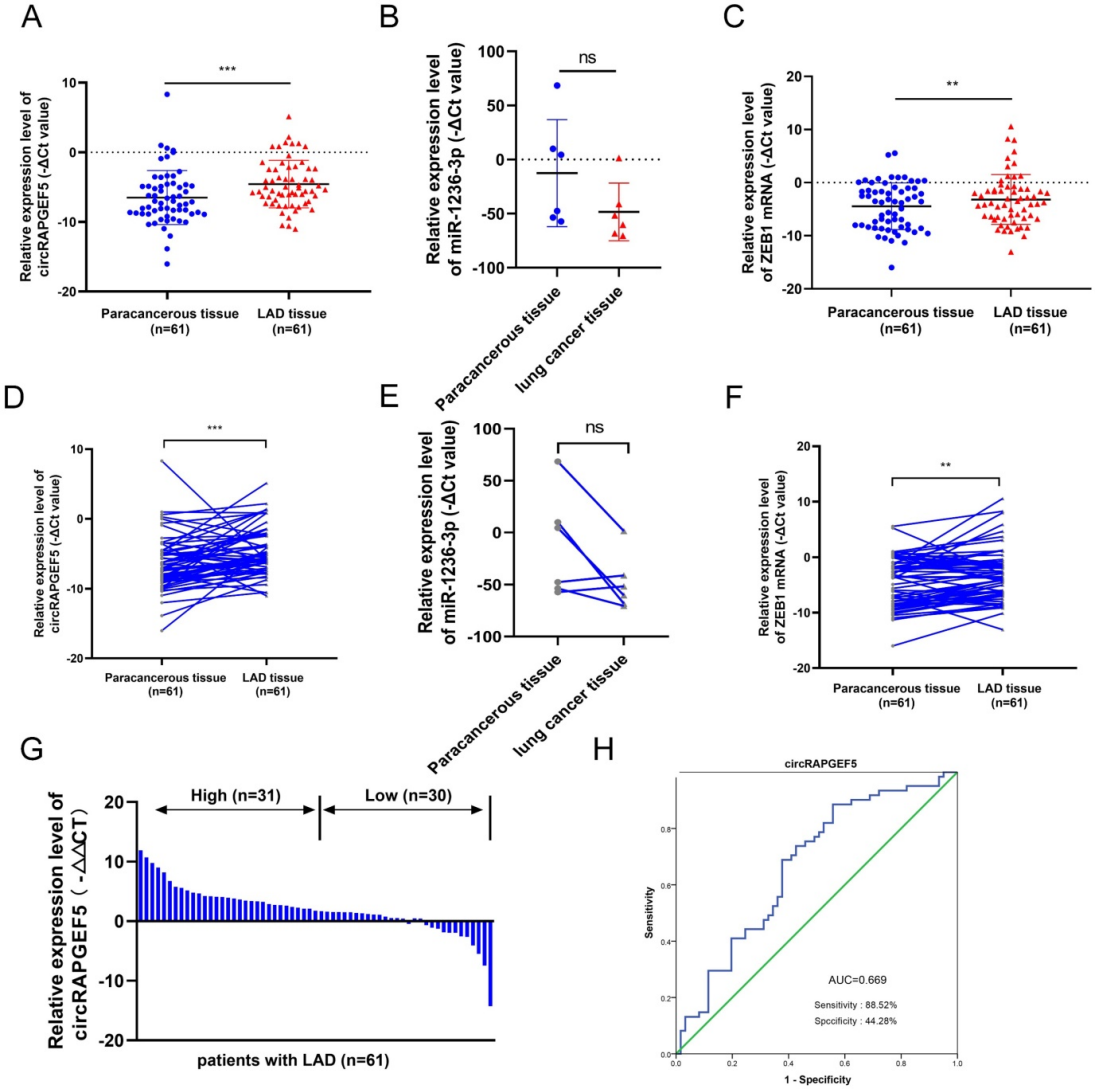
circRAPGEF5, miR-1236-3p and ZEB1 expression in LAD tissues. A, C, RT-qPCR was used to detect circRAPGEF5 (A) and ZEB1 (C) expression in 61 LAD tissues compared with paired paracancerous tissues. (B) Gene Expression Omnibus (GEO) datasets (GSE29248) was used to analyze miR-1236-3p expression between lung cancer tissues compared with paired paracancerous tissues. D, F, Sketch map of circRAPGEF5 (D) and ZEB1 (F) expression in 61 LAD tissues and paired paracancerous tissues. (E) Sketch map of miR-1236-3p expression in 6 lung cancer tissues and paired paracancerous tissues. (G) RT-qPCR analyzed circRAPGEF5 expression in LAD and paracancerous tissues. (H) Receiver operating characteristic (ROC) curve analysis to judge the recognition ability of circRAPGEF5 expression levels for patients with LAD in LAD tissues. ns: no significance, **P < 0.01, ***P < 0.001.

Compared to paracancerous tissues, circRAPGEF5 and ZEB1 were overexpressed in LAD tissues (Fig. 6A, C, D, F), and the expression level of miR-1236-3p in lung cancer was lower than that in paracancerous tissues, but there was no significant difference due to the small number of cases (Fig. 6B, E). According to the median circRAPGEF5 level, the 61 patients were divided into high (n = 31) and a low (n = 30) expression groups (Fig. 6G). We analyzed circRAPGEF5 expression and the clinical data of LAD patients and found that circRAPGEF5 was related to sex and lymph node metastasis (Table 1), but had no significant correlation with age, tumor size, T stage, M stage, and serum carcinoembryonic antigen (CEA). ROC curve analysis showed that circRAPGEF5 had the capability to diagnose LAD in patients (AUC value 0.669, sensitivity = 88.52%; specificity = 44.28%) (Fig. 6H).

### circRAPGEF5 participates in intercellular communication through exosomes

**Figure 7:**
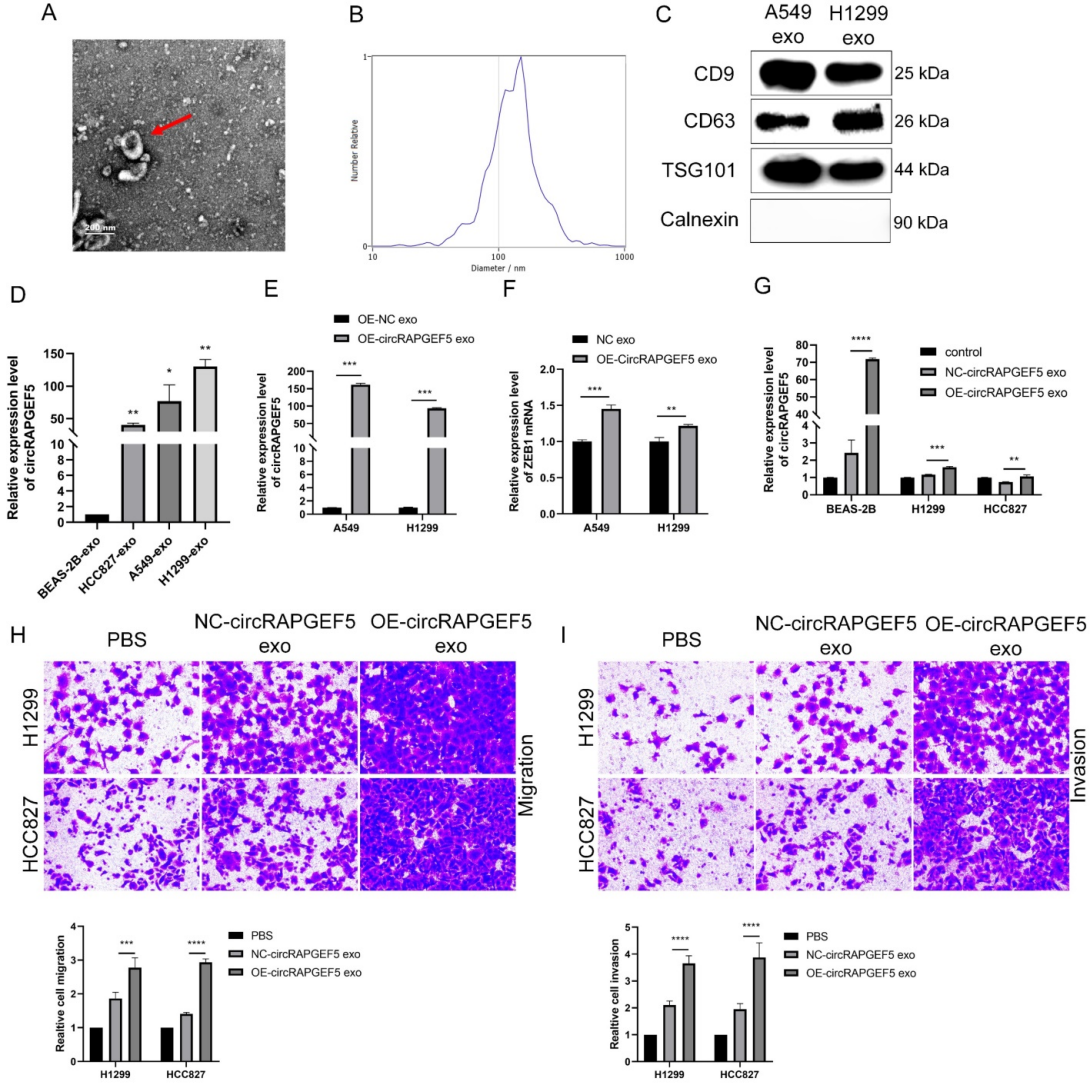
circRAPGEF5 participates in intercellular communication through exosomes. (A) Transmission electron microscope (TEM) of cell secreted exosomes. (B) The size of exosomes was detected by nanoparticle tracking analysis (NTA). (C) Specific markers of exosomes detected by western blotting. (D) Exosomal circRAPGEF5 expression from BEAS-2B cells and LAD cell lines was analyzed by RT-qPCR. E, F, The expression levels of circRAPGEF5 (E) and ZEB1 (F) in circRAPGEF5 overexpression and its control cells exosomes were analyzed by RT-qPCR. (G) circRAPGEF5 expression after co-culturing with circRAPGEF5 overexpression and its control cells exosomes (50 μg/ml) detected by RT-qPCR. H, I, Transwell assays detected migration (H) and invasion (I) of HCC827 and H1299 cells after co-culturing with circRAPGEF5 overexpression and its control cells exosomes (50 μg/ml). *P < 0.05, **P < 0.01, ***P < 0.001, ****P < 0.0001.

TEM results showed that exosomes were disc-shaped microvesicles (Fig. 7A). NTA analysis showed that the range of exosome diameter was 80‒200 nm (Fig. 7B). We tested CD9, CD63, TSG101, and calnexin protein expression to identify exosomes using western blotting (Fig. 7C). These experiments confirmed that the exosomes were successfully obtained.

Subsequently, we found that exosomal circRAPGEF5 was overexpressed in LAD cell lines, compared to exosomes derived from BEAS-2B cells (Fig. 7D). circRAPGEF5 and ZEB1 expression in OE-circRAPGEF5 exosomes increased significantly, especially in A549 cells (Fig. 7E, F). Therefore, A549 cells were used for the exosome preparation. Co-culture experiments with exosomes showed that circRAPGEF5 expression was upregulated after co-culturing with circRAPGEF5 high-expressing exosomes (Fig. 7G), and co-cultured with circRAPGEF5 high-expressing exosomes promoted migration and invasion (Fig. 7H, I). The above results indicated that circRAPGEF5 could be delivered through exosomes to promote LAD metastasis.

### Serum exosomal circRAPGEF5 acts as a promising biomarker of LAD

**Figure 8:**
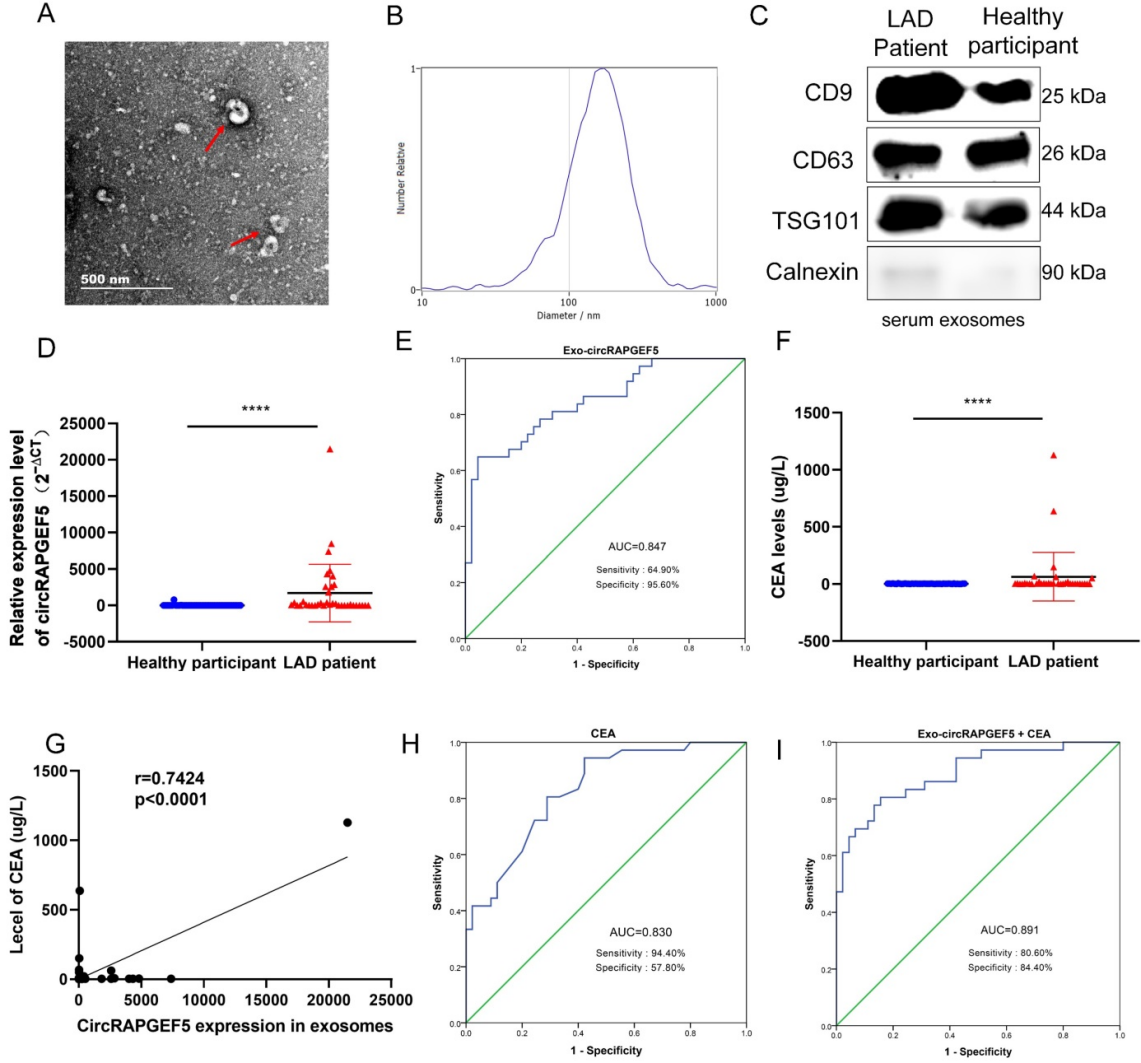
Serum exosomal circRAPGEF5 acts as a promising biomarker of LAD. (A) TEM of serum exosomes. (B) The size of exosomes was detected by NTA. (C) Specific markers of exosomes detected by western blotting. (D) Serum exosomal circRAPGEF5 expression from LAD patients and healthy participants was analyzed by RT-qPCR. (E) ROC curve analysis of Area Under Curve (AUC), sensitivity and specificity for serum exosomal circRAPGEF5 (Exo-circRAPGEF5) from LAD patients and healthy participants. (F) Serum CEA expression of LAD patients and healthy participants. (G) Correlation between circRAPGEF5 in serum exosomes and serum carcinoembryonic antigen (CEA) from LAD patients and healthy participants. (H) ROC curve analysis of AUC, sensitivity and specificity for serum CEA from LAD patients and healthy participants. (I) ROC curve analysis of AUC, sensitivity and specificity for serum CEA combined with serum Exo-circRAPGEF5 from LAD patients and healthy participants. ****P < 0.0001.

TEM results showed that exosomes were disc-shaped microvesicles, and the range of exosome diameter was 100‒250 nm; the expression of CD9, CD63, TSG101, and calnexin protein was tested to identify exosomes (Fig. 8A‒C). RT-qPCR revealed that serum exosomal circRAPGEF5 (Exo-circRAPGEF5) expression in LAD patients was significantly overexpressed compared to that in healthy participants (Fig. 8D). ROC curve analysis showed that serum Exo-circRAPGEF5 had excellent diagnostic ability (cut-off =11.8978, AUC = 0.847, sensitivity = 64.90%, and specificity = 95.60%) to distinguish LAD patients from healthy participants (Fig. 8E).

CEA, a biomarker for LAD diagnosis 22, was enhanced in the serum of patients with LAD (Fig. 8F), and there was a positive correlation between serum CEA and serum Exo-circRAPGEF5 expression in LAD (Fig. 8G). ROC curve analysis revealed that the AUC of serum CEA was 0.830, sensitivity was 94.40%, and specificity was 57.80% (Fig. 8H). The diagnostic ability of serum Exo-circRAPGEF5 for LAD patients was better than that of serum CEA. Furthermore, ROC curve analysis showed that serum Exo-circRAPGEF5 combined with serum CEA had better diagnostic performance (cut-off = 34.596, AUC = 0.891, sensitivity = 80.60%, and specificity = 84.40%) to distinguish LAD patients from healthy participants compared to single serum Exo-circRAPGEF5 or CEA (Fig. 8I). These results indicate that the combination of serum Exo-circRAPGEF5 and serum CEA may serve as a potential serum biomarker of LAD.

## Discussion

circRNAs have major functions in tumorigenesis and development, including renal cell carcinoma and breast, hepatic cellular, gastric, and lung cancer 23-27, but the basic mechanisms of circRNAs in these remain largely unclear. Due to their high stability and widespread presence in the human body 28, circRNAs may become an ideal biomarker. In this study, we found that circRAPGEF5 was overexpressed in LAD tissues and cells. Subsequently, we investigated the biological functions of circRAPGEF5 and found that circRAPGEF5 could promote the proliferation, migration, invasion, and EMT process of LAD cells *in vitro* and promote LAD cell growth *in vivo*. Therefore, we confirmed that circRAPGEF5 could promote the proliferation and metastasis of LAD.

Subsequently, we explored the molecular mechanism of circRAPGEF5. First, we predicted the relevant miRNAs using the TargetScan miRNA prediction website, and preliminarily identified miR-1236-3p, which was further verified using dual-luciferase reporter assay and RT-qPCR. We also confirmed that miR-1236-3p could reverse the tumor-promoting effect of circRAPGEF5. As a key transcription factor of EMT, ZEB1 can induce EMT and metastasis in a variety of cancer cells 29. We confirmed that circRAPGEF5 could upregulate ZEB1 expression, and ZEB1 knockdown could reverse the tumor-promoting effect of circRAPGEF5, indicating that ZEB1 is a major target of circRAPGEF5 to exert its function in LAD.

miR-1236-3p interacts with ZEB1 to inhibit the progression of LAD 20, we speculated that circRAPGEF5 could play a tumor-promoting effect through the miR-1236-3p/ZEB1 axis. We conducted a series of experiments to verify this, and the results showed that miR-1236-3p could combine with ZEB1 to reduce ZEB1 expression. In addition, the upregulation of miR-1236-3p decreased circRAPGEF5-upregulated ZEB1 expression. In contrast, inhibition of miR-1236-3p could rescue the downregulation of ZEB1 caused by circRAPGEF5 knockdown. The above results indicated that circRAPGEF5 could promote the proliferation and metastasis of LAD via the miR-1236-3p/ZEB1 axis.

Further analysis revealed that compared with paracancerous tissues, circRAPGEF5 in LAD tissues was evidently increased. Analysis of the clinical characteristics revealed that circRAPGEF5 overexpression was related to lymph node metastasis. These results indicate that circRAPGEF5 is related to LAD metastasis and is consistent with the cytological results.

Exosomes play a significant role in intercellular communication and participate in the occurrence and progression of tumors 30, 31. However, research on exosomal circRNAs in the LAD is still unclear. We found that circRAPGEF5 was overexpressed in LAD cell exosomes. Subsequently, we also found that the exosomes derived from OE-circRAPGEF5 cells had higher levels of circRAPGEF5. Based on these results, we conducted co-culture experiments with exosomes and found that circRAPGF5 expression increased after incubation with circRAPGEF5 high-expressing exosomes by RT-qPCR, and the migration and invasion ability was enhanced after incubation with circRAPGEF5 high-expressing exosomes. These results demonstrated that circRAPGEF5 could be transmitted between cells through exosomes to promote LAD metastasis.

CEA is an acid glycoprotein that exists on the surface of cancer cells and can be detected in various body fluids 32, 33. however, it lacks specificity and sensitivity 34. Therefore, it is of great significance for the prevention and treatment of LAD to explore simple, fast, highly sensitive, and specific biomarkers. Assessment of circRNA expression signatures in exosomes is a promising tool for tumor research and clinical diagnosis 35. For example, a high level of circ-PNN in serum exosomes may be a potential biomarker for colorectal cancer 36. In this study, patients with LAD had higher levels of serum Exo-circRAPGEF5 than healthy participants. The diagnostic ability of serum Exo-circRAPGEF5 (AUC = 0.847) for LAD patients was better than that of serum CEA (AUC = 0.830). Then, we combined serum Exo-circRAPGEF5 with serum CEA and found that the AUC was as high as 0.891, sensitivity = 80.60%, and specificity = 84.40%. The results indicated that the combination of serum Exo-circRAPGEF5 and serum CEA may be a potential serum biomarker of LAD. Unlike tissue biopsy, serum exosomal detection only involves drawing blood, which is a less invasive method. Therefore, combined with the high expression and high stability of circRNAs, we believe that the detection of serum exosomal circRAPGEF5 may be a promising biomarker of LAD.

In summary, we proved that circRAPGEF5 played a significant role in the proliferation and metastasis of LAD by the miR-1236-3p/ZEB1 axis. In addition, circRAPGEF5 could affect the function of recipient cells through exosomal transmission, thereby promoting metastasis of LAD, and serum exosomal circRAPGEF5 may be a promising biomarker to identify LAD.

## Supplementary Material

Supplementary figures and tables.

## Figures and Tables

**Table 1:** Correlation of circRAPGEF5 expression with clinical characteristics

Parameters	Patients (n=61)	circRAPGEF5		*P* value
		High expression (n=31)	Low expression (n=30)	
Gender				0.0151*
male	29	10 (34.48%)	19 (65.52%)	
female	32	21 (65.63%)	11 (34.37%)	
Age (years)				0.5000
<60	25	14 (56.00%)	11 (44.00%)	
≥60	36	17 (47.22%)	19 (52.78%)	
T stage				0.2381
T1~2	57	28 (49.12%)	29 (50.88%)	
T3~4	3	3 (100.00%)	0 (0.00%)	
X^#^	1	0 (0.00%)	1 (100.00%)	
N stage				0.0261*
N0	52	23 (44.23%)	29 (55.77%)	
N1~3	9	8 (88.89%)	1 (11.11%)	
M stage				>0.9999
M0	60	30 (50.00%)	30 (50.00%)	
M1	1	1 (100.00%)	0 (0.00%)	
Tumor size (cm)				0.8213
≤2	34	18 (52.94%)	16 (47.06%)	
>2	26	13 (50.00%)	13 (50.00%)	
X^#^	1	0 (0.00%)	1 (100.00%)	
CEA (μg/L)				0.3305
≤5	44	24 (54.55%)	20 (45.45%)	
>5	15	6 (40.00%)	9 (60.00%)	
X^#^	2	1 (50.00%)	1 (50.00%)	

The median expression level of circRAPGEF5 was used as the cut-off value. *: *P* < 0.05; ^#^: “X” indicated that the tumor could not be evaluated or measured for the clinical characteristics, and these patients were not included in the statistical test. Abbreviation: CEA, carcinoembryonic antigen.

## Abbreviations

Lung adenocarcinoma: LAD
Epithelial-mesenchymal transition: EMT
circular RNA: circRNA
Ribonuclease R: RNase R
competing endogenous RNA: ceRNA
real-time quantitative PCR: RT-qPCR
fetal bovine serum: FBS
overexpressed circRAPGEF5: OE-circRAPGEF5
knockdown circRAPGEF5: KD-circRAPGEF5
Transmission electron microscope: TEM
Nanoparticle tracking analysis: NTA
Cell Counting Kit-8: CCK-8
Horseradish peroxidase: HRP
enhanced chemiluminescence: ECL
wild-type: WT
mutated: Mut
carcinoembryonic antigen: CEA
receiver operator characteristic: ROC
area under the receiver operator characteristic curve: AUC
exosomal circRAPGEF5: Exo-circRAPGEF5
