# Supplementary material for: CircRAPGEF5 Promotes the Proliferation and Metastasis of Lung Adenocarcinoma through the miR-1236-3p/ZEB1 Axis and Serves as a Potential Biomarker
Source: Int J Biol Sci. 2022 Feb 28;18(5):2116–31. doi: 10.7150/ijbs.66770 (PMC8935214; doi:10.7150/ijbs.66770)
Supplement: Supplementary file 1 — Supplementary figures and tables. [file ijbsv18p2116s1.pdf]

Supplementary Table S1. The primer Sequences of genes

Primer ID	Primer Sequences (5’-3’)	Product length （bp）
circRAPGEF5 F	GCCTCTCATTCCTGCCAGAG	115
circRAPGEF5 R	TCTTGATAGAGTCGCAGATGTTAGA	
ZEB1 F	GCCTGAGTCCTCTGTTTCA	128
ZEB1 R	GCTCTTCTGCACTTGGTTG	
β-actin F	GACGGCTACCCGATCTCGGCAT	176
β-actin R	ACGGCTTTCCAGCGCATCCGCA	
U6 F	AGAGAAGATCGGCATGGTTACTG	213
U6 R	ACCTGAGCGCATTGTC CGAGG	
GAPDH F	GACGCGTGGGAACATGAGACA	157
GAPDH R	ATGACCTGGACGGTCGAGCGC	

Supplementary Table S2. Top ten significantly different circRNAs in lung adenocarcinoma

Probe ID	*P* value	Fold chande	Regulation	circRNAs
**ASCRP3001831**	**0.004858**	**6.955763**	**up**	**hsa_circ_0001681**
ASCRP3008913	0.002065	2.951749	up	hsa_circ_0079557
ASCRP3006277	0.024133	2.94732	up	hsa_circ_0000897
ASCRP3007951	0.007272	2.789349	up	hsa_circ_0000872
ASCRP3009875	0.016353	4.93492	down	hsa_circ_0049271
ASCRP3010445	0.023019	4.912611	down	hsa_circ_0000788
ASCRP3006087	0.013213	4.804215	down	hsa_circ_0030569
ASCRP3008380	0.040182	4.607752	down	hsa_circ_0053944
ASCRP3009800	0.030668	4.428522	down	hsa_circ_0061817
ASCRP3004018	0.022251	4.084411	down	hsa_circ_0008689

Supplementary Figure S1.

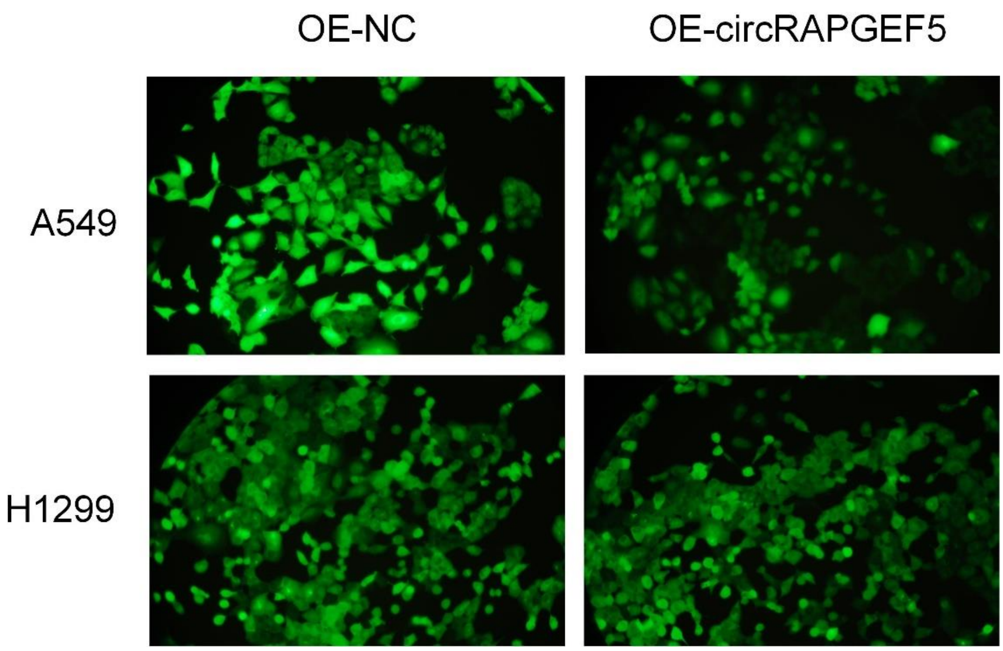

Figure S1. A fluorescence microscope was used to observe the cell fluorescent signal after lentivirus transfection (OE-circRAPGEF5: circRAPGEF5 overexpression; OE-NC: negative control of OE group; MOI = 10).

Supplementary Figure S2.

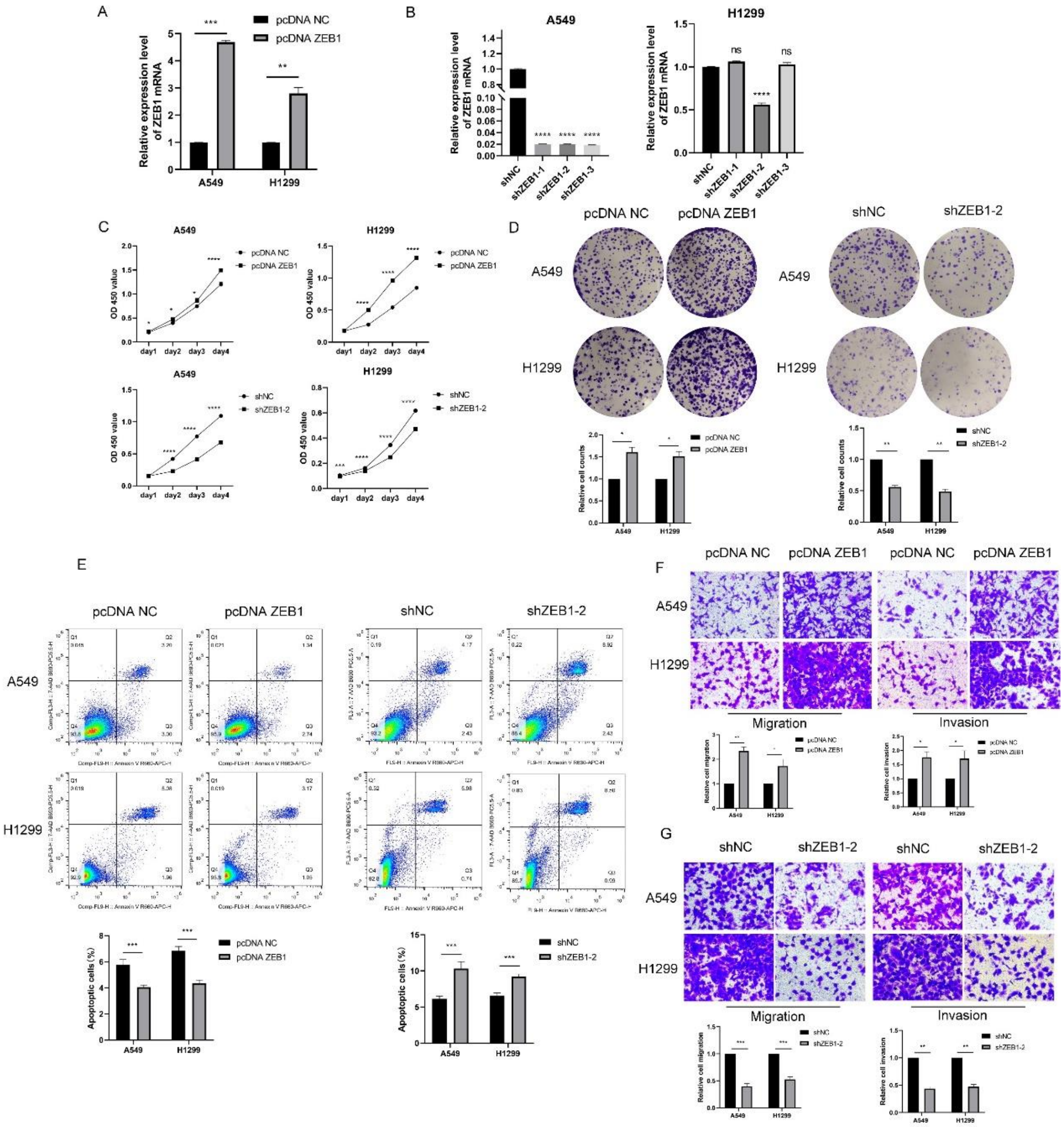

Figure S2. ZEB1 promotes proliferation, migration and invasion of LAD cell lines. A, B, qPCR analysis of ZEB1 expression after ZEB1 overexpression (500 ng/12-well plates) (A) and knockdown (50 nM) (B) in A549 and H1299 cells. (C) CCK-8 assays detected proliferation of ZEB1 overexpression and knockdown in LAD cells. (D) The colony formation assays detected colony formation ability of ZEB1 overexpression and knockdown in LAD cells. (E) Flow cytometric analyzed apoptosis rates in LAD cells with ZEB1 overexpression and knockdown. F, G, Transwell analysis detected migration and invasion ability in LAD cells with ZEB1 overexpression (F) and knockdown (G). ns: no significance, **P* < 0.05, ***P* < 0.01, ****P* < 0.001, *****P* <

0.0001.
